# C-855-08. Survey on Comfort with Burn Injury Management Among Providers in a Remote and Rural Setting

**DOI:** 10.1093/jbcr/irag033.092

**Published:** 2026-04-06

**Authors:** Darby Little, George Ho, Margarita S Elloso, Shahriar Shahrokhi

**Affiliations:** University of Toronto, Toronto, ON; University of Toronto, Toronto, ON; McMaster University, Hamilton, ON; McMaster University, Hamilton, ON

## Abstract

**Introduction:**

Rural and remote communities often rely on primary care providers to complete the initial management and referral of burn injuries. This study aimed to assess frontline providers’ comfort with the initial assessment and management of burn injuries in a rural and remote setting.

**Methods:**

A cross-sectional survey was distributed to primary and wound care providers across 10 healthcare facilities in a large rural and remote region between August and December 2024. Self-reported comfort levels with 15 aspects of burn care were assessed using a five-point Likert scale. Wilcoxon rank-sum tests were used to compare survey responses across practice characteristics.

**Results:**

Fifty-nine providers participated, including family physicians (44%), nurse practitioners (20%), emergency physicians (10%), and others. While 90% saw at least one burn injury per year, only 42% saw more than five. Most respondents reported comfort with tetanus prophylaxis (72%) and burn first aid (66%). However, fewer felt comfortable assessing burn size (31%) or depth (27%), initiating fluid resuscitation (27%), or applying the American Burn Association guidelines for burn center transfer (24%). Providers managing five or more burns annually reported significantly greater comfort across multiple domains (*p*<.05). No significant differences were found between early- and late-career providers.

**Conclusions:**

This study demonstrates that providers working in a rural and remote region reported limited comfort with burn size and depth assessment, initiating fluid resuscitation, and applying burn center referral guidelines – important skills for initial injury management and referral. These findings highlight a need for system-level supports and educational resources for providers in regions remote from specialized burn centers.

**Applicability of Research to Practice:**

Ninety percent of primary and wound care providers see at least one burn injury per year in a region that is geographically removed from specialized burn care. Providers reported limited comfort with multiple key facets of initial burn care, including the application of burn center referral guidelines. These results point to opportunities for greater engagement and the development of supports that address the unique challenges of rural and remote burn care.

**Funding for the study:**

N/A.

